# Proteomics approaches to long COVID: status and outlooks

**DOI:** 10.1093/lifemedi/lnad023

**Published:** 2023-06-19

**Authors:** Xiao Liang, Yingrui Wang, Tiannan Guo

**Affiliations:** iMarker lab, Westlake Laboratory of Life Sciences and Biomedicine, Key Laboratory of Structural Biology of Zhejiang Province, School of Life Sciences, Westlake University, Hangzhou 310024, China; Institute of Basic Medical Sciences, Westlake Institute for Advanced Study, Hangzhou 310024, China; Research Center for Industries of the Future, Westlake University, Hangzhou 310030, China; Center for Infectious Disease Research, Westlake University, Hangzhou 310024, China; iMarker lab, Westlake Laboratory of Life Sciences and Biomedicine, Key Laboratory of Structural Biology of Zhejiang Province, School of Life Sciences, Westlake University, Hangzhou 310024, China; Institute of Basic Medical Sciences, Westlake Institute for Advanced Study, Hangzhou 310024, China; Research Center for Industries of the Future, Westlake University, Hangzhou 310030, China; Center for Infectious Disease Research, Westlake University, Hangzhou 310024, China; iMarker lab, Westlake Laboratory of Life Sciences and Biomedicine, Key Laboratory of Structural Biology of Zhejiang Province, School of Life Sciences, Westlake University, Hangzhou 310024, China; Institute of Basic Medical Sciences, Westlake Institute for Advanced Study, Hangzhou 310024, China; Research Center for Industries of the Future, Westlake University, Hangzhou 310030, China; Center for Infectious Disease Research, Westlake University, Hangzhou 310024, China

While we are facing continuous threat from SARS-CoV-2, which causes the global COVID-19 pandemic, the post-acute sequelae of SARS-CoV-2 infection (PASC) emerge as new concerns, which may have affected more than half of the COVID-19 survivors [1]. More commonly known as long COVID, PASC refers to a wide range of post-COVID symptoms that appear around 3 months after infection and last for a long time and cannot be explained by an alternative diagnosis [2]. Since its first case report in August 2020, over 2,000 long COVID papers have been published. Most studies are based on epidemiological approaches to analyze susceptible population. Risk factors for long COVID-19 include female, severe symptoms during COVID-19, and the presence of basic diseases such as diabetes and hypertension. A series of studies used clinical indicators extracted from electronic hospital records to investigate alternated molecules in long COVID, and found that long COVID patients maintained an inflammatory or hypercatabolic state. Additionally, high levels of viremia and the presence of auto-immunity might be contributing factors to long COVID. These findings describe the clinical manifestation of long COVID, but the molecular mechanisms accounting for the symptoms remain a mystery. Proteomics could systematically analyze the molecular changes throughout the disease course of long COVID, even before symptoms appear. So far, proteomics has been widely applied in COVID-19 [3] but relatively few in long COVID. In this research highlight, we enumerate a few proteomics-based long COVID studies, describe their enabling techniques, summarize their key findings, discuss the limitations, and propose several future research directions.

Proteomics-based long COVID studies discussed in this research highlight were performed with two major approaches, mass spectrometry (MS) and Olink (Table 1). In MS-based approaches, proteins are firstly extracted from serum/plasma samples and enzymatically digested into peptides. A liquid chromatography system then separates the peptide mixture, and the eluted peptide aliquots are ionized for the first round of MS measurement (MS1). Thereafter, a subset of the peptides is fragmented for the second round of MS measurement (MS2). The MS results were annotated as peptide and protein matrices using bioinformatics software and the MS signal intensities were used for quantification. Key advantages of MS-based proteomics include its capability to characterize thousands of proteins (deep coverage) across large numbers of samples (high throughput). For example, 1,311 proteins from over 150 serum samples were identified in Chen et al.’s study. The depth of serum proteome could be improved to over 3,000 proteins when combined with adequate fractionation techniques. Additionally, MS-based proteomics was especially suitable for discovery-driven studies, such as Pretorius et al.’s work that we discuss below. Moreover, MS is also applicable for targeted analysis by selecting peptides of pre-defined mass-to-charge signals for MS2 analysis, which has been a demonstrated technique to validate prioritized COVID-19 markers [10].

**Table 1.** Summary of approaches to proteomics-based long COVID studies

Approach	Publication	Sample type	Protein ID	Strengths
MS-based	Chen et al. [4]	Serum	1,311	Deep coverage; high throughout; feasible for both targeted and untargeted analysis
	Pretorius et al. [5]	Plasma and amyloid deposits	Not provided	
	Captur et al. [6]	Plasma	91	
Olink	Zhao et al. [7]	Plasma	92	Accurate; sensitive; easy to implement
	Vijayakumar et al. [8]	BAL	435	
	Su et al. [9]	Plasma	454	

BAL, bronchoalveolar lavage.

Other than MS, an emerging approach, Olink proteomics, was applied for the remaining studies. Olink is based on a targeted immunoassay that recognizes the proteins of interest with specially designed antibody pairs linked with unique DNA oligonucleotides. The complementary regions of the oligos are therefore in proximity, hybridized, and further extended to form double strands, which could be amplified and quantified using polymerase chain reaction. Olink proteomics takes advantage of antibody recognition and nucleotide amplification, therefore is both accurate and sensitive, and especially suited for analyzing cytokines of low concentrations from microliters of blood. An additional advantage of Olink proteomics is its easy implementation, in that proteins do not have to be prepared as peptides and could be analyzed by sequencers. Zhao et al. used Olink Target 96 Inflammation Panel that contains targeted characterization of 92 inflammatory biomarkers and four internal controls [7]. Su et al. and Vijayakumar et al. used four additional panels and characterized 454 and 435 proteins, respectively. Olink currently can characterize maximally 3,072 proteins, and can also be designed for discovery proteomics assays. Additionally, Su et al. additionally identified 192 PBMC surface proteins based on Chromium cell surface protein labeling, a similar but simpler approach that uses single antibodies for recognition and conjugated oligonucleotide barcodes for quantification [9]. This technique could be optimized to achieve a higher identification number. Beyond these, SOMAscan is an alternative non-MS technology that uses designated SOMAmer (Slow Off-rate Modified Aptamer) to characterize specific proteins. The bound SOMAmers are measured using standard DNA analysis techniques. SOMAscan currently can characterize utmost 7000 proteins and was used to analyze patient behaviors during the acute phase of COVID-19.

**Figure 1.**
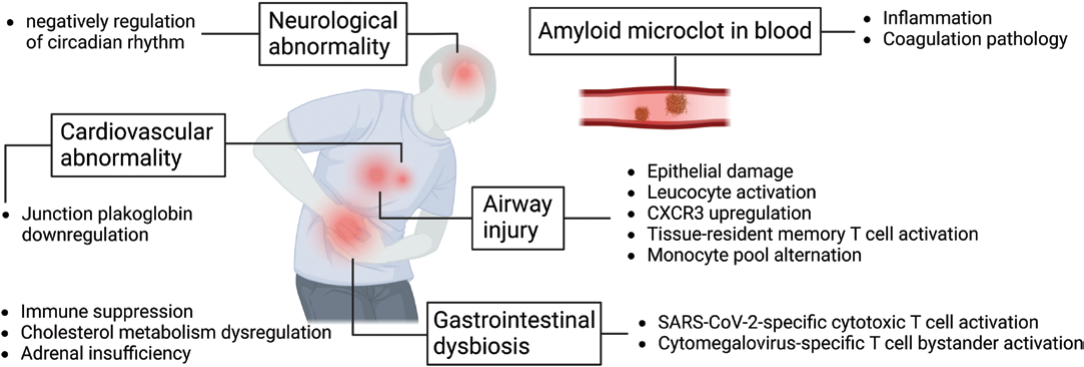
**Mechanistic clues of proteomics-based long COVID studies.** The frames denote long COVID symptoms and the entries denote altered proteins or functions in long COVID.

Proteomics-based long COVID studies were mostly realized by assessing the body fluids post COVID. Vijayakumar et al. characterized bronchoalveolar lavage (BAL) proteomes from 19 post-COVID-19 patients at 3 to 6 months after hospital discharge (Fig. 1) [8]. A major finding is the epithelial damage in long COVID airways, characterized by the enrichment of myoglobin, caspase-3, and epithelial cell adhesion molecule. Additionally, COVID-19 survivors with upregulated C-X-C Motif Chemokine Receptor 3 (CXCR3) also exhibited prolonged epithelial and extracellular matrix damage. These early clues prompted the researchers to analyze immune cell identities and find activated tissue-resident T memory cells as well as alternated monocyte pool in the post-COVID-19 airways. Another study by Chen et al. characterized the serum proteomes of 60 and 58 COVID-19 survivors at 6 and 12 months after the disease, respectively [4]. The survivors exhibited immune suppression shown by a list of downregulated immunological pathways, compared to the healthy individuals. A persisting cholesterol metabolism dysregulation was also observed in the survivors, characterized by the downregulated apolipoprotein A1, a main component of high-density lipoprotein l (HDL). Notably, HDL is protective for the endothelial layer and its downregulation may lead to endothelial cell damage, a hallmark during COVID-19 infection. Cardiovascular function and blood coagulation-related proteins were also disturbed despite the survivors’ blood biochemistries maintained normal. Pretorius et al. analyzed the persisting amyloid deposits in the long COVID plasma [5] and found that these microclots contained elevated inflammatory proteins such as serum amyloid A and antiplasmin, compared to the plasma from non-COVID-19 individuals. Same findings were observed in acute COVID-19, suggestive of a persisting clotting pathology and the related pathway regulation since COVID-19 onset. These evidence suggest that the molecular dysregulation in the COVID-19 survivors was both pervasive and persistent several months after recovery, especially regarding their immunological functions.

Subdividing COVID-19 survivors based on their symptoms could strengthen the connection between proteomics characteristics and long COVID phenotypes. Su et al. characterized plasma proteomes in around 300 post-COVID individuals [9] and found that, patients reporting neurological abnormalities exhibited elevated proteins that negatively regulate the circadian rhythm pathway. Moreover, patients with over three long COVID symptoms might experience adrenal insufficiency. Su et al. further analyzed the single-cell transcriptome signatures of peripheral blood mononuclear cells in long COVID, enabling a molecular stratification of the survivors into four immune endotypes. Another study by Zhao et al. compared the inflammatory protein markers in 22 post-COVID-19 patients with long COVID symptoms and 22 that were asymptomatic [7]. They found that COVID-19 survivors, whether symptomatic or asymptomatic, exhibited persistent inflammation. Only one protein, C-X-C Motif Chemokine Ligand 10 (CXCL10), was differentiated and upregulated in long COVID patients, bearing potential as a predictive marker for long COVID. Alternatively, using a targeted MS approach, Captur et al. characterized 91 inflammatory biomarkers in the plasma samples from 54 COVID-19 patients [6]. Based on the protein expression at the time of infection and the 12-month-stage symptom persistence of these patients, they built a classification model that can predict long COVID at the time of infection.

Proteomics-based long COVID studies should also be considered in light of limitations. Mechanistic findings such as persistent inflammation might be attributed to basic diseases or comorbidities that are irrelevant to COVID-19 or long COVID. These confounding factors are hard to be excluded, due to the relatively small cohorts of only tens of recruited patients. On the other hand, the relatively high cost for either MS-based or Olink proteomics makes it impractical for large-scale population-based analysis to reduce confounding effects. Additionally, circulating proteins are less indicative compared to medical imaging, when assessing inflammation and injury status of specific organs.

Prospectively, clinical characteristics of long COVID symptoms should be continuously manifested to bring the strength of proteomics into full play. Also, multi-layered proteomics to analyze not only protein expression but also its post-translational modification and structural changes might deepen our mechanistic understanding, considering that the delayed onset characteristics of long COVID may result from microenvironmental factors not exhibited genetically. Moreover, incorporating metabolomics analysis would help understand the hypercatabolic states and cholesterol metabolism dysregulation in long COVID. Beyond investigating the mechanisms, proteomics-based translational studies are of great promise as well. Protein markers derived from easily accessible biofluids, such as urine and saliva, could be exploited as risk factors for an early prediction of long COVID. The serum or PBMC protein markers, on the other hand, could be considered as drug targets for long COVID treatment.
